# Preoperative magnetic resonance imaging of anal fistulas with scrotal extension: a retrospective study

**DOI:** 10.3389/fsurg.2023.1224931

**Published:** 2023-07-20

**Authors:** Duc Tan Vo, Truc Thi Thuy Nguyen, Nam Hoang Nguyen, Linh Thi Thuy Nguyen, Thien Thi Thanh Nguyen, Chien Cong Phan

**Affiliations:** ^1^ Department of Diagnostic Imaging, University Medical Center, Ho Chi Minh City, Vietnam; ^2^ Department of Radiology, University of Medicine and Pharmacy at Ho Chi Minh City, Ho Chi Minh City, Vietnam

**Keywords:** anal fistula, scrotal extension, magnetic resonance imaging, goodsall’s rule, midline rule

## Abstract

**Introduction:**

This study aimed to elucidate the magnetic resonance (MR) characteristics of anal fistulas extending to the scrotum, and the applicable rules, and to correlate MR features with surgical findings.

**Methods:**

We conducted a retrospective study in 150 consecutive patients with anal fistulas extending into the scrotum, who were diagnosed and underwent surgery at University Medical Center Ho Chi Minh City between January 2017 and April 2022. MR findings were evaluated and compared with surgical findings using Cohens kappa coefficient (k) with a 95% confidence interval.

**Results:**

150 patients (mean age 37.6 ± 10.9 years) with 166 fistulas, including 150 anal fistulas with scrotal extension. Most fistulas were low transsphincteric (80.0%, 120/150 patients). There was a strong agreement for primary tract classification and detecting the location of internal openings between MRI and surgical findings with *k* = 0.83 (0.780.87) and *k* = 0.89 (0.85 0.93) (*p*<0.001), respectively. There is a significant correlation between the location of internal openings and the type of fistula (*p*<0.05). Low transsphincteric fistulas were predominant in the anterior group (103/122 patients vs. 10/19 patients), while in the posterior group, it was more common in the high transsphincteric fistulas (7/19 patients vs. 14/122 patients), and the intersphincteric fistulas (1/19 patients vs. 5/122 patients); and the suprasphincteric fistulas were only seen in the posterior group (1 patient).

**Conclusion:**

Anal fistulas with scrotal extension are exceptions to Goodsalls rule. Albeit long-tract fistulas, most are low transsphincteric and have anterior internal openings.

## 1. Introduction

An anal fistula is a common disease in the anorectal region, defined as an abnormal tract connecting the anorectal canal and the perineal skin. Anal fistulas affect approximately 0.01% of the population. They are more common in men in their fourth decade, which is two- to four-fold compared to women (1–3).

In some cases of males with anal fistulas, the fistulas can extend into the scrotum. The presentations are variable in those cases. The scrotum usually presents with redness, swelling, pain, and pus discharge from the external opening, while anal symptoms may be absent or minimal (4–6). Such presentations may be confusing with acute scrotal diseases such as infection, abscess of the scrotum, testis, or urethral diverticulum, or even malignancy. In addition, other conditions may be encountered along the median raphe over the perineum and scrotum, which are called anterior perineal sinuses, those from urogenital origins, Cowper's gland cysts, benign neoplastic conditions, epidermal inclusion cysts, and pilonidal disease (6–8). With a high index of suspicion, careful palpation can reveal the fistulous tract between the external opening into the scrotum and the internal opening into the anal canal. Involvement of the scrotum in anal fistulas can lead to diagnostic confusion, aggressive interventions, and reconstructive challenges (6, 9, 10).

Goodsall's rule for predicting the trajectory of anal fistulas is popular; however, the long-tract anterior fistula, which may include fistulas with scrotal extension, is an exception to this rule (10–12). On the other hand, over the decades, the research literature has shown the importance of the midline as the location of the internal opening of all anal fistulas. Midline Rule had a superior predictive accuracy of up to 95%, regardless of the location of the external opening. The Midline rule predicts the location of the internal opening with a PPV of 71% (vs. 49% using Goodsall's rule) when describing the origin of anterior-based anal fistula, including fistulas with scrotal extension (13). Magnetic resonance (MR) is extremely useful in these cases thanks to its capability to provide precise anal canal anatomy and the relation of the fistula to perineal structures. It allows precise definition of the fistulous track and identification of secondary fistulas or abscesses, helping the surgeons avoid side effects (such as fecal incontinence) and decreasing the incidence of recurrence (14, 15).

According to our best knowledge, this is the first study about the role of MR in the preoperative evaluation of anal fistulas with scrotal extension. We conducted this study to elucidate the MR characteristics of anal fistulas extending to the scrotum, the rules applicable to them, and to correlate MR features with surgical findings.

## 2. Materials and methods

### 2.1. Patients

**Figure 1:**
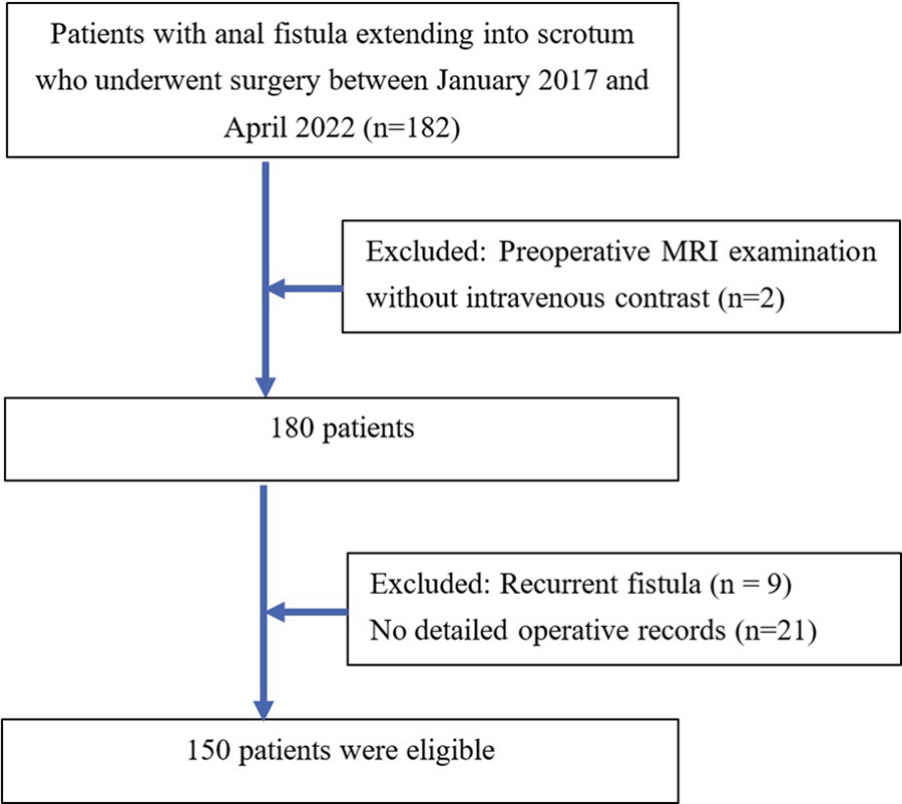
Diagram of patient inclusion and exclusion.

A total of 150 consecutive patients with anal fistulas extending into the scrotum who were diagnosed and underwent surgery at the University Medical Center, Ho Chi Minh City between January 2017 and April 2022 were included in this study. All included patients underwent preoperative MRI examination with intravenous contrast. The exclusion criteria were (a): patients having recurrent fistula, or (b) their operative records were not detailed (Figure 1).

### 2.2. Magnetic resonance protocol

MR imaging was performed on either a 1.5 Tesla (MAGNETOM Avanto, Siemens Healthineers, Germany) or 3 Tesla (MAGNETOM Verio, Siemens Healthineers, Germany) system using six-channel body phased-array coil for pelvic imaging and no special patient preparation was required.

The MR protocol consisted of the following pulse sequences: sagittal T2-weighted (T2W) turbo spin-echo (TSE), oblique coronal T2W TSE with fat saturation (FS), and oblique axial T2W TSE with the imaging planes oriented orthogonal and parallel to the axis of the anal canal, respectively.

The parameters for these T2W sequences were repetition time (TR) 3,000–5,000 ms, echo time (TE) 75–85 ms, field of view (FOV) 20–25 cm, turbo factor (TF) 17, slice thickness 3–4 mm, intersection gap 0.6–0.7 mm, matrix 320 × 256 (for the sagittal and coronal plane) or 320 × 320 (for the axial plane), number of signals acquired (NSA) 2. The T1-weighted (T1W) TSE FS with gadolinium enhancement (post-contrast) (0.2 ml/kg) was obtained in three planes (axial, coronal, and sagittal). The parameters for post-contrast T1W TSE FS are TR 600–700, TE 10–12 ms, FOV 20–25 cm, TF 3, slice thickness 3–4 mm, intersection gap 0.6–0.7 mm, matrix 320 × 256 (for the coronal plane) or 320 × 320 (for the sagittal and axial plane), NSA 2.

### 2.3. Image analysis

We retrospectively reviewed the patient's medical records, including their operative records and MR findings. The MR images were analyzed by two radiologists who had more than five years of experience in MR fistulography. We classified anal fistulas in the study according to Park's classification, which includes intersphincteric, transsphincteric, suprasphincteric, and extrasphincteric fistulas. We further classified transsphincteric fistulas as high or low as follows: high transsphincteric fistulas pass through the upper half of the external anal sphincter, whereas low transsphincteric fistulas traverse the lower half (16, 17).

**Figure 2:**
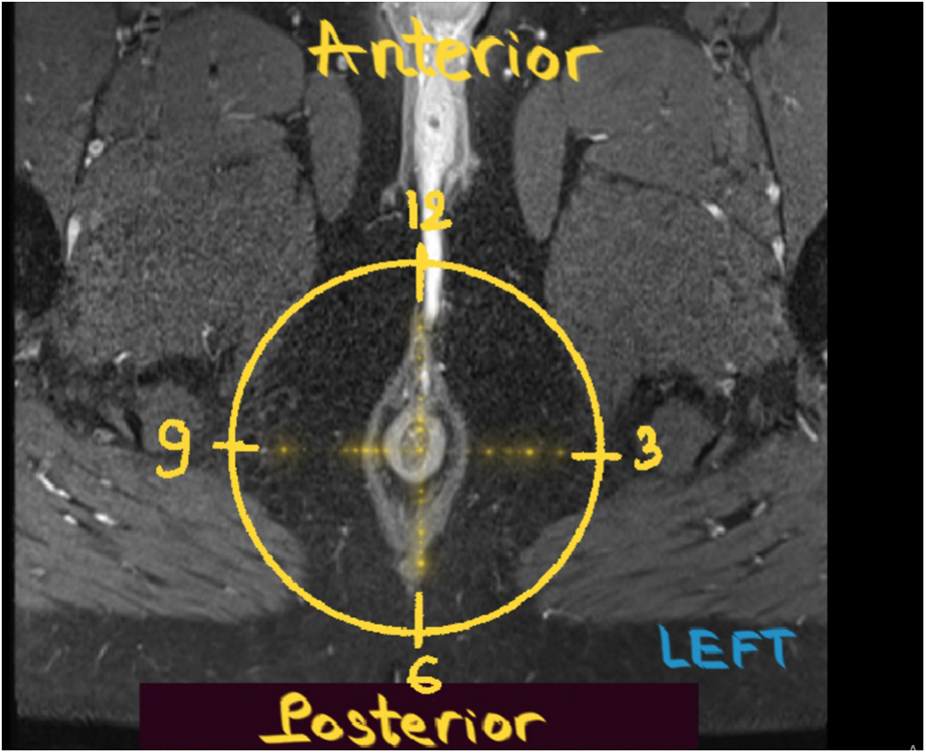
Anal clock. Post-contrast axial T1W FS MR image of the male perineum obtained with the patient in the supine position shows the anal clock diagram.

We defined the scrotal extension of the anal fistula (with or without external openings as well as the distance between the external opening and anal verge) using the physical examination results reported in the operation records. On MR, internal openings were detected when the tract terminated in the intersphincteric space (16, 18). The location of the internal openings was described according to anal clock positions (Figure 2). Openings at the 10, 11, 12, 1, and 2 o'clock positions were classified as anterior, while those at the 3, 4, 5, 6, 7, 8, and 9 o'clock positions were classified as posterior. A one-hour difference between MR images and surgery was considered acceptable.

### 2.4. Statistical analysis

Statistical analysis was conducted using STATA version 16 (STATA Corp., Texas, USA). Obtained data were presented as mean and standard deviation (with normal distribution) or median and interquartile range (with skewed distribution), ranges, numbers, and ratios. Numbers and ratios were analyzed using Fisher's exact test. A *p*-value < 0.05 was considered statistically significant.

MR findings were compared with surgical findings using Cohen's kappa coefficient (*k*) with a 95% confidence interval. The degree of agreement was interpreted as follows: ≤0 as no agreement, 0.01–0.20 as none to slight, 0.21–0.40 as fair, 0.41–0.60 as moderate, 0.61–0.80 as substantial, and 0.81–1.00 as almost perfect agreement.

## 3. Results

### 3.1. Patient characteristics

150 male patients having anal fistulas with scrotal extension were eligible for the study. The mean age at surgery was 37.6 ± 10.9 years (range 14–79 years). The age group of 30–50 was the most common (62.0%), followed by the age group of under 30 (26.0%) and over 50 (12.0%). Most fistulas had histological findings of non-specific inflammation (98.7%, 148/150), and only 2 patients were due to tuberculosis. The mean duration from MR acquisition to surgery was 1.5 ± 1.8 days (range 0–15 days).

### 3.2. MR images analysis

There was a total of 150 patients with 166 fistulas, including 136 patients with one fistula (with scrotal extension), 12 patients with two fistulas (one fistula with scrotal extension and another fistula), and 2 patients with three fistulas (one fistula with scrotal extension and two other fistulas).

#### 3.2.1. Scrotal extension (with and without external opening in the scrotum)

The external opening was detected in 142/150 (94.7%) patients with 159 external openings. 129 patients (86.0%) had one, 10 patients (6.7%) had two, 2 patients (1.3%) had three, and 1 patient (0.7%) had four external openings. In the remaining 8 patients without external openings detected, the presence of scrotal involvement was recorded based on surgical reports. Including these 8 cases, the total number of locations where scrotal involvement took place was 167. Among the aforementioned 167 locations, we noticed a left-side predominance (38.9%, 65/167), followed by midline (33.5%, 56/167) and right-side (27.6%, 46/167).

#### 3.2.2. Primary tract

Each of the 150 patients had only one fistula-in-ano extending to the scrotum. According to Parks classification, most of the fistulas were classified as low transsphincteric (80.0%, 120/150 patients), followed by high transsphincteric (14.7%, 22/150 patients), intersphincteric (4.0%, 6/150 patients), and suprasphincteric fistula (1.3%, 2/150 patients). There was a strong agreement for primary tract classification between MR and surgical findings with a kappa value of 0.83 (95% CI: 0.78–0.87) (*p* < 0.001), which is demonstrated in Table 1.

**Table 1:** Agreement between MR and surgery in the classification of primary tract according to the parks classification.

Surgery MRI	Low trans	High trans	Inter	Supra	Total
Low trans	114	5	1	0	120
High trans	1	19	1	1	22
Inter	0	0	6	0	6
Supra	0	0	0	2	2
Total	115	24	8	3	150

Low trans, low transsphincteric fistula; High trans, high transsphincteric fistula; Inter, intersphincteric fistula; Supra, suprasphincteric fistula.

Data are presented as numbers of primary tracts. Kappa value: 0.83 (95% CI: 0.78–0.87), *p* < 0.001.

#### 3.2.3. Internal opening

**Figure 3:**
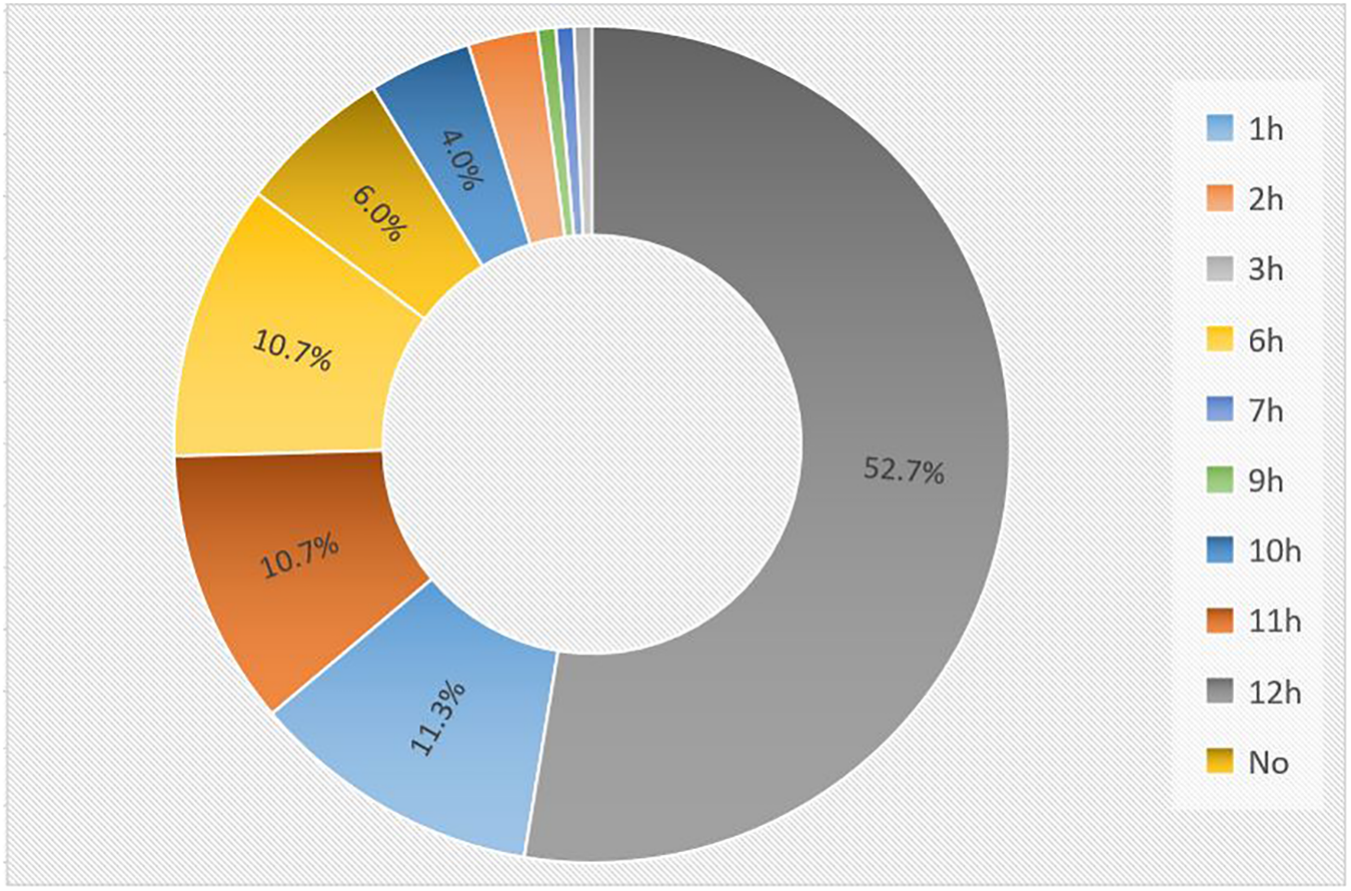
The distribution of internal openings in MR imaging. The most common location of the internal openings was from 11 o'clock to 1 o'clock (74.7%), followed by 6 o'clock (10.7%). The remaining positions were less common.

141 internal openings were detected in 150 patients. The most common location of the internal openings was from 11 o'clock to 1 o'clock (74.7%) at the level of the dentate line, followed by 6 o'clock (10.7%). The remaining positions were less common (Figure 3). There was good agreement in detecting the location of internal openings between MR and surgical findings with a kappa value of 0.89 (95% CI: 0.85–0.93) (*p* < 0.001), which is shown in Table 2.

**Table 2:** Agreement between MR and surgery in detecting the location of internal openings.

Surgery MRI	1 h	2 h	3 h	6 h	7 h	8 h	9 h	10 h	11 h	12 h	No i.o	Total
1h	12	0	0	0	0	0	0	0	0	4	1	17
2h	0	3	1	0	0	0	0	0	0	0	0	4
3h	0	0	1	0	0	0	0	0	0	0	0	1
6h	0	0	0	13	0	0	0	0	0	0	3	16
7h	0	0	0	0	1	0	0	0	0	0	0	1
8h	0	0	0	0	0	0	0	0	0	0	0	0
9h	0	0	0	0	0	0	1	0	0	0	0	1
10h	0	0	0	0	0	0	3	3	0	0	0	6
11h	0	0	0	1	1	0	0	1	12	1	0	16
12h	5	0	0	0	0	0	0	0	2	71	1	79
No i.o	1	0	0	0	0	1	0	0	1	1	5	9
**Total**	18	3	2	14	2	1	4	4	15	77	10	150

i.o, internal opening.

Data are presented as numbers of internal openings. Kappa value: 0.89 (95% CI: 0.85–0.93), *p* < 0.001.

We observed a significant correlation between the location of the internal opening (anterior or posterior to the transverse anal line) and the fistula type (Table 3). Low transsphincteric fistulas accounted for the majority of fistulas having anterior internal openings (84.4%). In the posterior group, the percentages of low transsphincteric and non-low-transsphincteric fistulas (including intersphincteric, high transsphincteric, and suprasphincteric fistulas) were roughly comparable (52.6% and 47.4%, respectively). However, it was more common in the high transsphincteric fistulas (7/19 patients vs. 14/122 patients), the intersphincteric fistulas (1/19 patients vs. 5/122 patients); and the suprasphincteric fistulas were only seen in the posterior group (1 patient).

**Table 3:** The relation between the location of internal openings and the fistula type.

Fistula type Location of i.o	Low trans	High trans	Inter	Supra	Total
Anterior group	103	14	5	0	122
Posterior group	10	7	1	1	19
No i.o	7	1	0	1	9
Total	120	22	6	2	150

Low trans, low transsphincteric fistula; High trans, high transsphincteric fistula; Inter, intersphincteric fistula; Supra, suprasphincteric fistula; i.o, internal opening.

Data are presented as numbers of patients. There was a significant correlation (*p* < 0.05).

The mean distance from the external opening to the anal verge was 6.2 ± 2.5 cm (range 3–12 cm). The mean distances from the external opening to the anal verge of the anterior and posterior internal opening group were 6.2 ± 2.5 cm (range 3–12 cm) and 6.4 ± 2.3 cm (range 3–10 cm), respectively. No statistical significance was found between the two groups (*p* > 0.05).

## 4. Discussion

Occasionally, an anal fistula can extend into the scrotum, causing pain, edema, and pus discharge from the external opening and making clinicians confused with other scrotal disorders. Our study found that anal fistulas with scrotal extension occurred mainly in men around the age of 40, which is consistent with previous studies (2, 3).

We observed that patients with anal fistulas extending into the scrotum had one to three fistulas, and zero to four external openings, but the majority finding was one fistula extending into the scrotum with one external opening.

According to Park's classification, anal fistulas can be classified into four types based on their relationship to anal sphincters: intersphincteric, transsphincteric, suprasphincteric, and extrasphincteric in which the intersphincteric fistulas were the most common (70%) (19). In our study, the most prominent fistula type was low transsphincteric (76.7%). This is in accordance with the results of Araki's study, which reported that low transsphincteric was the most prevalent categorization (82.6%) among anal fistulas extending to the scrotum. Because the Colles' fascia which is composed of loose connective tissue overlays the anterior portion of the superficial external sphincter muscle, anterior low transsphincteric fistulas can penetrate the external sphincter and directly enter this fascia. Meanwhile, high transsphincteric and suprasphincteric fistulas with posterior internal openings often curve anteriorly in the subcutaneous fat tissue of the ischiorectal fossa instead of penetrating the Colles' fascia.

**Figure 4:**
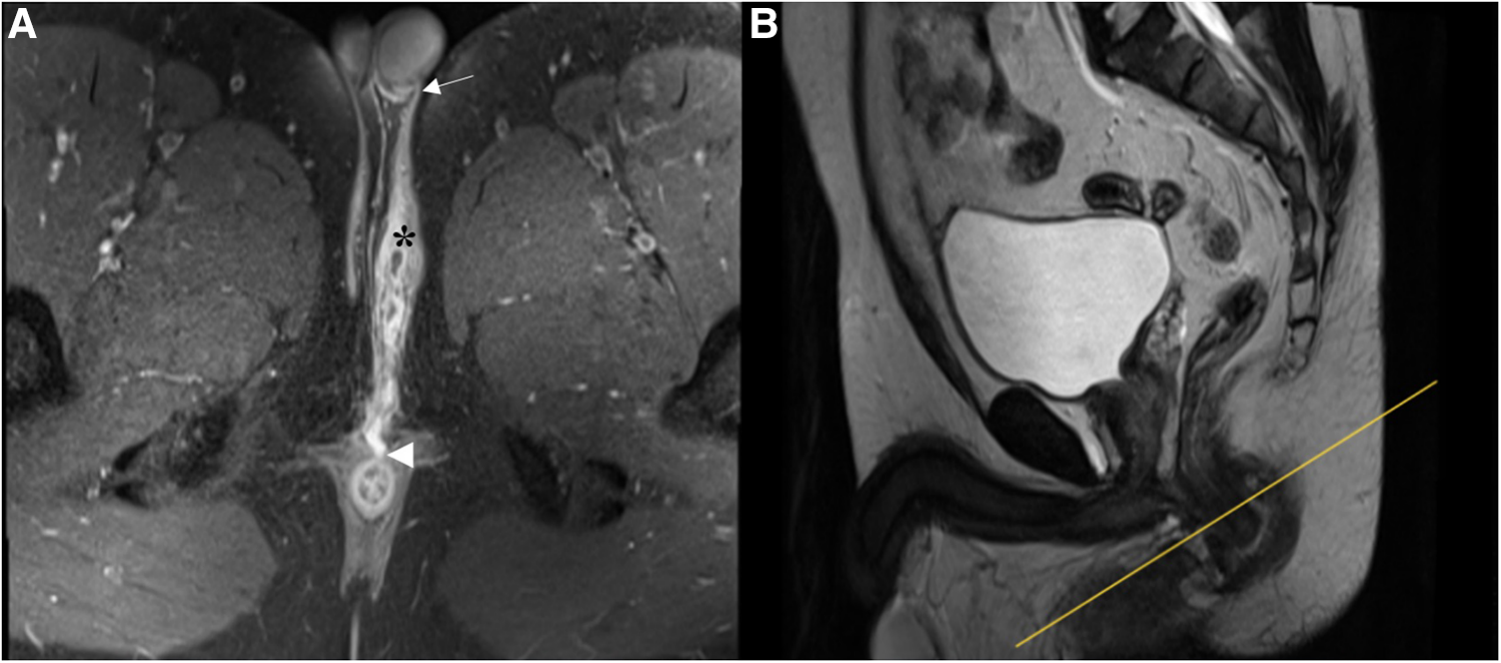
A low transsphincteric fistula extending to the left scrotum. (**A)** Post-contrast FS axial T1-weighted image shows a fistula (asterisk) extending into the left scrotum (arrow) and opening radially into the anterior wall of the anal canal at 12 o'clock position (arrowhead). (**B**) Sagittal T2-weighted image shows the corresponding location (yellow line) where the fistula penetrating the external sphincter. The fistula traversed the lower half of the anal canal and was classified as low transsphincteric.

**Figure 5:**
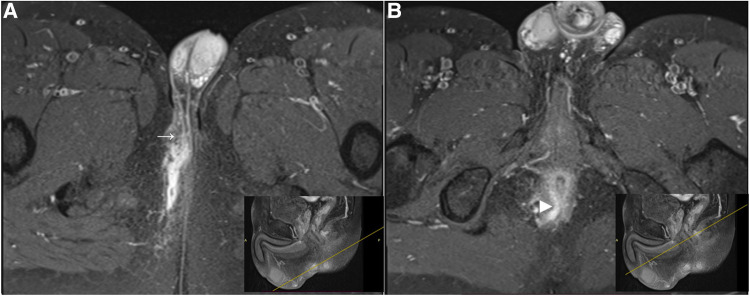
A high trannsphincteric fistula extending into the right scrotum. Post-contrast FS axial T1-weighted images show (**A**) an anal fistula extending into the right scrotum (arrow) with a curved track (**B**) which opens into the posterior wall of the anal canal at 6 o'clock position (arrowhead). The corresponding location (yellow line) where the fistula penetrates the external sphincter. The fistula traverses the upper half of the anal canal and we classified it as a high transsphincteric fistula.

In the present study, there was a strong agreement for primary tract classification between MR imaging and surgical findings, qualifying MR as a valuable modality in the preoperative classification of anal fistulas. On MR, high transsphincteric fistulas passed through the upper half of the external sphincter, whereas low transsphincteric fistulas traversed the lower half (Figures 4, 5). The above-mentioned MR classification of transsphincteric fistulas is acceptable and simple to apply, especially when each part of the external sphincter is not clearly defined. The proposed classification has been used for deciding between non-sphincter-preserving and sphincter-preserving procedures (11, 17). There were instances when surgery and MR disagreed on categorizing transsphincteric fistulas as high or low. This may be due to the difference between surgery and MRI in the selection of anatomical landmarks. Surgeons divided transsphincteric fistulas into high and low based on the parts of the external sphincter being penetrated: a fistula was considered high transsphincteric if it penetrated the deep external sphincter, while one penetrating the superficial or subcutaneous external sphincter was deemed low transsphincteric (9).

The location of scrotal involvement in our study was most common in the left scrotum (38.9%), followed by the midline (33.5%) and the right scrotum (27.6%). This is consistent with the study of Araki et al. (9), which also reported that the left scrotum was the most common site of scrotal extension. A study of 367 patients (16) demonstrated that most intersphincteric and low transsphincteric fistulas had external openings located less than 3 cm from the anal verge, whereas the majority of high transsphincteric, suprasphincteric and extrasphincteric fistulas had their external orifices situated more than 3 cm from the anal verge. By contrast, in our work, although all external openings were more than 3 cm away from the anal verge, the fistulas were mostly low transsphincteric. Consequently, the scrotal extension can be considered a predictive sign for simple fistulas instead of complicated ones as some previous classifications assumed (20), thus fistulotomy could be done safely without any risk of incontinence.

**Figure 6:**
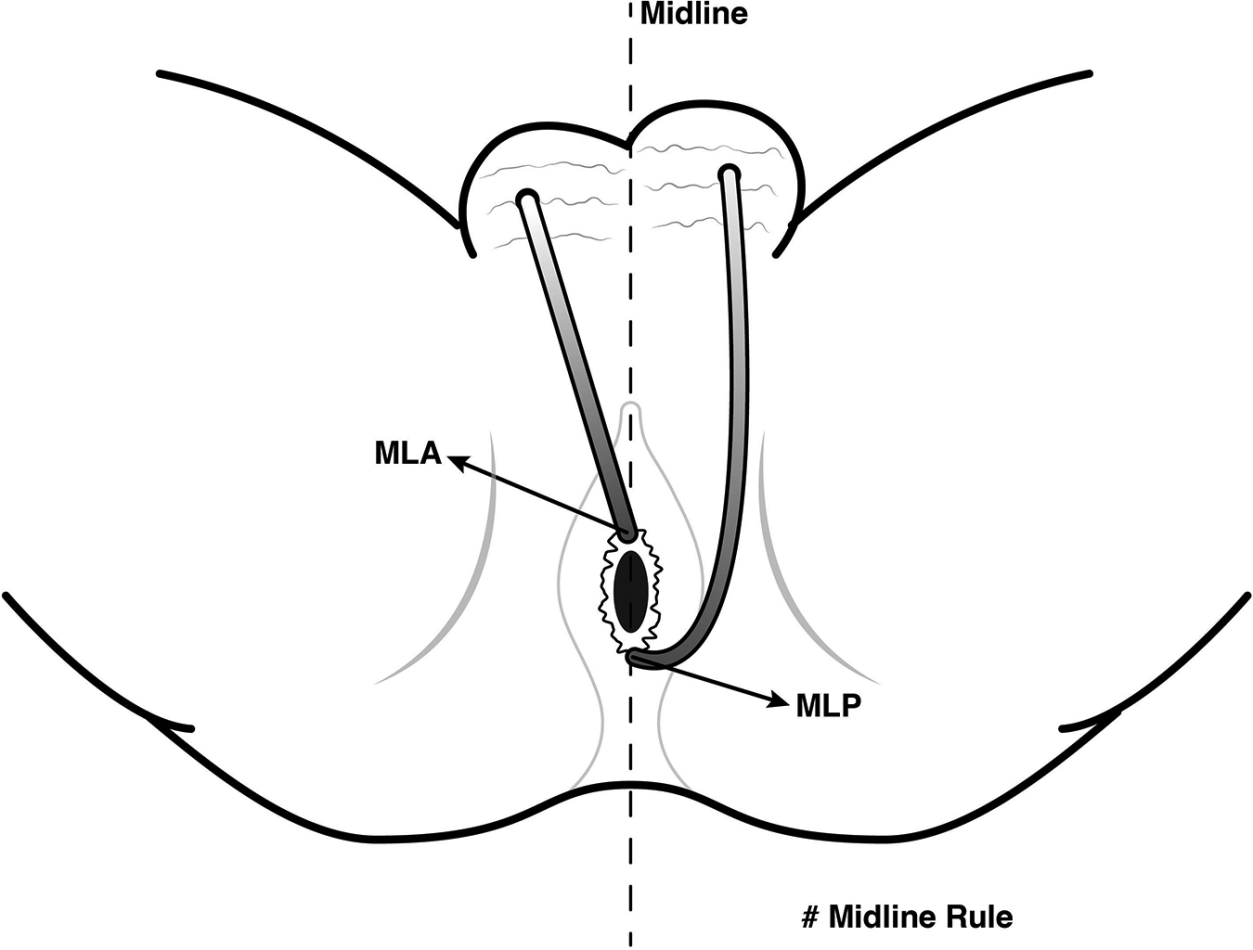
Midline rule for anal fistulas with scrotal extension. The Midline rule stated the midline crypt was the primary internal origin of all anal fistulas regardless of the external opening location, especially those with anterior-based external openings, including fistulas with scrotal extension. *MLA, midline anterior; MLP, midline posterior.*

Proper identification of the internal opening is an integral part of fistula surgery to avoid recurrence (21). On MR, we may not be able to visualize the location of the fistula opening into the anal canal mucosa, where its signal is as high as that of the fistula. As a result, some authors have suggested looking for the area of maximal intersphincteric inflammation to detect the internal opening (15, 16). The good agreement between MR and surgery in detecting the location of internal openings illustrated in our study supports the notion that MR is a reliable modality for the preoperative assessment of internal openings in anal fistulas with scrotal extension. The most common location of the internal openings was from 11 o'clock to 1 o'clock (74.7%) at the mid-portion of the anal canal, corresponding to the level of the dentate line on surgery followed by 6 o'clock (10.7%). Both midline anterior and midline posterior positions accounted for 85.4% of the total internal openings in our study. This is in accordance with the Midline rule mentioned in previous reports, which stated the midline crypt was the primary internal origin of all anal fistulas regardless of the external opening location, with up to 95% accuracy (13) (Figure 6). In comparison with Goodsall's rule, the Midline rule more accurately predicted the natural course of anal fistulas, especially those with anterior-based external openings, including fistulas with scrotal extension (13, 22).

**Figure 7:**
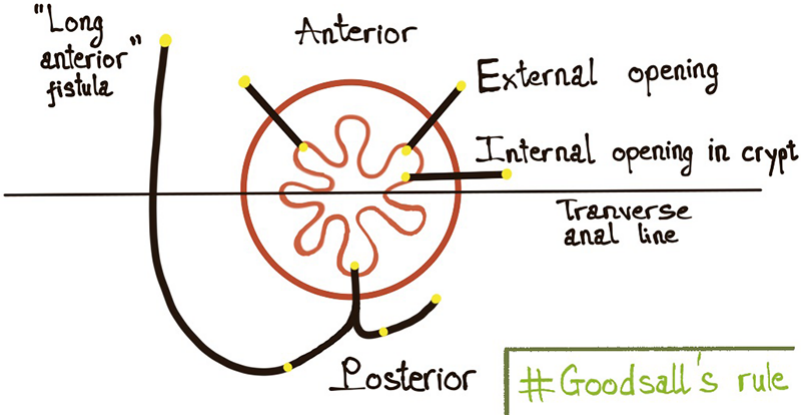
Goodsall's rule.

We observed a significant correlation between the location of the internal opening (anterior or posterior to the transverse anal line) and the fistula type (*p* < 0.05). This is consistent with the previous reports (9, 23) which showed that the majority of anal fistulas extending into the scrotum were low transsphincteric fistulas with an anterior internal opening. Also, high transsphincteric and suprasphincteric fistulas usually had posterior internal openings. This contributes to the prediction that fistulas with posterior internal openings are more complex than ones with anterior internal openings. Goodsall's rule states that if the external opening is anterior to the transverse anal line, the fistula will penetrate radially and open into the anterior wall of the anal canal provided it is less than 3 cm from the anal verge, or else it will open in the midline posteriorly. When lying more than 3 cm from the anal verge, the anterior fistulas may have a curved track, which is similar to the posterior fistulas (4, 24) (Figure 7). This study shows that the anal fistula with scrotal extension was an exception to Goodsall's rule: though the external openings were more than 3 cm from the anal verge, most fistulas had an anterior internal opening (86.5%, 122/141 patients) (Figure 4). Anal fistulas that do not comply with Goodsall's rule have the risk of creating iatrogenic false tracks and openings when being probed (13, 25). Accordingly, it is not secure for surgeons to solely rely on Goodsall's rule to identify the internal openings in cases where the tracts extend to the scrotum. Also, because the tracks are long and may involve male urinary or genital structures, preoperative MR is necessary to improve surgical outcomes. In these instances, MR is a valuable and reliable modality for preoperative evaluation, which eventually helps reduce the risk of complications (fecal incontinence, urinary tract damage, and sexual dysfunction) as well as recurrence.

Though the external openings are more than 3 cm from the anal verge, most anal fistulas with scrotal extension are low transsphincteric and have anterior internal openings, making them exceptions to Goodsall's rule. On the other hand, they are consistent with the Midline rule, so we suggest surgeons utilize the Midline rule when dealing with fistulas extending to the scrotum. We recommend performing magnetic resonance in fistulas with scrotal extension thanks to its excellent performance in classifying the primary tract and detecting the internal opening, which are major contributors to surgical prognosis.

## Data Availability

The raw data supporting the conclusions of this article will be made available by the authors, without undue reservation.
